# ALK Positive Lung Cancer: Clinical Profile, Practice and Outcomes in a Developing Country

**DOI:** 10.1371/journal.pone.0160752

**Published:** 2016-09-16

**Authors:** Vanita Noronha, Anant Ramaswamy, Vijay M Patil, Amit Joshi, Anuradha Chougule, Subhadha Kane, Rajiv Kumar, Arvind Sahu, Vipul Doshi, Lingaraj Nayak, Abhishek Mahajan, Amit Janu, Kumar Prabhash

**Affiliations:** 1 Department of Medical Oncology, Tata Memorial Hospital, Mumbai, India; 2 Department of Pathology, Tata Memorial Hospital, Mumbai, India; 3 Department of Radiology, Tata Memorial Hospital, Mumbai, India; University of Nebraska Medical Center, UNITED STATES

## Abstract

**Objectives:**

To evaluate the performance and treatment profile of advanced EML4—ALK positive Non-small cell lung cancer (NSCLC) patients in a developing country with potentially restricted access to Crizotinib.

**Materials and Methods:**

A retrospective analysis of advanced ALK positive NSCLC patients who were treated from June 2012 to September 2015 was conducted. The primary goal was to evaluate outcomes of advanced ALK positive NSCLC in our practice and examine the logistic constraints in procuring Crizotinib.

**Results:**

94 patients were available for analysis. 21 (22.3%) patients were started on Crizotinib upfront, 60 (63.8%) on chemotherapy, 10 (10.6%) on Tyrosine kinase inhibitors (in view of poor PS) and 3 (3.2%) patients were offered best supportive care. Reasons for not starting Crizotinib upfront included symptomatic patients needing early initiation of therapy (23.3%), ALK not tested upfront (23.3%) and financial constraints (21.9%). 69 patients (73.4%) received Crizotinib at some stage during treatment. Dose interruptions (> 1 week) with Crizotinib were seen in 20 patients (29%), with drug toxicity being the commonest reason (85%). Median Progression free survival (PFS) on first line therapy for the entire cohort was 10 months, with a significant difference between patients receiving Crizotinib and those who did not ever receive Crizotinib (10 months vs. 2 months, p = 0.028). Median Overall Survival (OS) was not reached for the entire cohort, with 1 year survival being 81.2%. Patients with an ECOG Performance Status (PS) of >2 had a significantly reduced PFS compared to patients with PS < = 2 (1.5 months vs. 11 months, p< 0.001). 47 patients with financial constraints (68.1%) received Crizotinib completely free via various extramural support schemes.

**Conclusion:**

A majority of our ALK positive NSCLC patients were exposed to Crizotinib through the help of various support mechanisms and these patients had similar outcomes to that reported from previously published literature.

## Introduction

The discovery of the Anaplastic lymphoma kinase (ALK) gene in non-small cell cancers (NSCLC) and the subsequent use of Crizotinib against this subset of NSCLC has been one of the success stories of precision medicine in lung cancer [1–4]. The presence of the driver EML4 –ALK fusion also identifies a category of NSCLC patients who are characterized clinicopathologically by relatively younger age, non-smokers or light smokers, and a mucinous, cribriform or signet–ring cell subtype of adenocarcinoma [5–7].

After initial single arm studies and retrospective analysis showed clinical benefit from Crizotinib [3,8,9], FDA approval was granted based on the results of a Phase III study, which showed higher response rates (65% vs. 20%)and PFS benefit (7.7 mo. vs. 3mo) for Crizotinib as second line therapy in ALK positive NSCLC [10]. Benefit in first line status was confirmed by the PROFILE 1014 trial, which showed a PFS improvement of Crizotinib (10.9 mo. vs. 7 mo.) over standard 1^st^ line palliative chemotherapy, cementing its status as the first line option in ALK positive NSCLC [4].

However, the potentially prohibitive cost of Crizotinib in a resource constrained setting and additional costs associated with testing for ALK fusion make it mandatory to examine all aspects of therapy before deciding on course of treatment. It is essential to evaluate treatment patterns in such a scenario, where extramural mechanisms are routinely required for optimization of treatment in an economically challenged population. Hence, we carried out a retrospective analysis at our centre to investigate the treatment patterns and outcomes in Indian ALK-positive NSCLC patients, with an emphasis on procurement, use, tolerance and outcomes with Crizotinib.

## Materials and Methods(“Fig 1”)

### Patient selection (“Fig 1”)

Patients from June 2012 to September 2015 were selected for this analysis subject to the following criteria (all criteria to be fulfilled)–

1. Advanced NSCLC
2. Planned for palliative treatment
3. Presence of ALK fusion reported as positive, either by immunohistochemistry (IHC) or break-apart Fluorescence In situ Hybridization (FISH)

**Fig 1:**
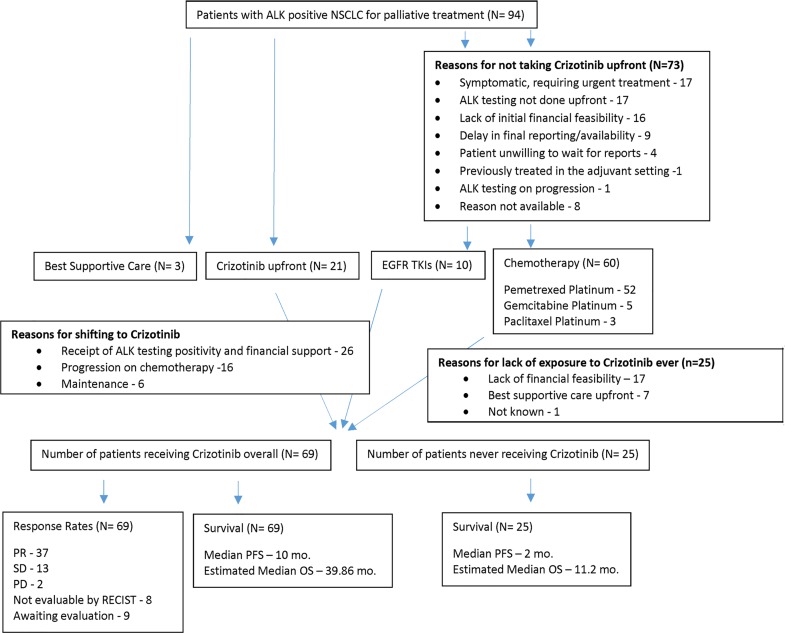
Study Outline.

The details of these patients were obtained from the prospective lung cancer audit database that is maintained in the department of medical oncology. The lung cancer audit is an Institutional Ethics Committee (IEC) approved observational protocol, is registered with the Clinical Trials Registry India (registration number: CTRI/2013/01/003335) and patients sign a written informed consent prior to their information being recorded as part of the lung cancer audit.

#### Pretreatment evaluation

Patients underwent a complete history and physical examination, and routine blood testing (complete hemogram, renal and liver function test) prior to therapy. Demographic data including smoking status and tobacco use was collected. Tumor staging was performed by a contrast enhanced computed tomography of the chest and upper abdomen(CECT) or Whole body FDG PET–CET.

Detection of ALK fusion was by either or both of the following methods:

1. FISH—Formalin-fixed, 4 μm thick, paraffin-embedded cell blocks were used to evaluate the ALK genetic status with FISH. The slides were deparaffinized, dehydrated, immersed in 0.2 N HCl for 20 min, then washed and incubated in pretreatment solution at 80°C. In this test, the 5^’^ and 3^’^ ends of the ALK gene are labelled with red and green fluorescent probes respectively. ALK positivity or rearrangement is reported if the signals are not touching (split). The FISH analysis was performed with the ‘Abbot Molecular’ platform, according to manufacturer instructions. 100 nuclei were scored to determine the final percentage of ALK positivity. A cut-off of 15% was used to denote samples as positive or negative for ALK.
2. IHC—IHC for ALK was performed with the monoclonal antibody D5F3 (Ventana Medical Systems, Tucson, AZ, USA).

#### Treatment and follow up

Patients were started on therapy based on age, comorbidities, ECOG Performance status (PS), disease burden requiring emergent initiation of therapy, availability of ALK reports and feasibility for Crizotinib (elucidated separately). Patients were started on either of the following treatments upfront–

1. Chemotherapy
2. Crizotinib 250mg PO twice daily
3. Oral Tyrosine Kinase Inhibitor (prior to availability of ALK status and in view of poor ECOG PS, i.e. >2)
4. Best supportive care

Patients underwent routine blood investigations, including complete hemogram and biochemistry prior to each cycle of chemotherapy and every monthly or 2 monthly if on Crizotinib. Additionally, ECGs were monitored on Crizotinib at frequent intervals. Dose reduction was considered as per standard criteria.

Assessment of radiological response was done every 2–3 months and at symptomatic progression. Chemotherapy or targeted therapy was discontinued at disease progression, intolerable side effects, or patient decision. Tumor response was evaluated by RECIST 1.1 criteria. The adverse events during this period were evaluated in accordance with the Common Terminology Criteria for Adverse Events version 4.02 (CTCAE v 4.02). At progression, further therapy was considered based on standard recommendations.

#### Evaluation of pattern of use of Crizotinib

Patients were divided into those who received Crizotinib upfront, received Crizotinib at a later time or were never exposed to Crizotinib. Potential reasons for not administering Crizotinib upfront were retrieved. Patients receiving Crizotinib at some later point of their treatment course were examined for reasons of this shift in therapy. The source of financing for patients on Crizotinib was also reported.

#### Statistical analysis

SPSS version 16 was used for analysis. Descriptive statistics was performed. Median value with interquartile range was provided for continuous variables. Progression free survival (PFS) was calculated in months from the date of start of Crizotinib till the date of progression on Crizotinib. Patients who had not progressed at the time of last follow up were censored. Overall survival (OS) was calculated in months from the date of start of Crizotinib till the date of death. Patients who had not died at the time of last follow up were censored. Kaplan Meier method was used for time to event analysis. Log rank test was used for univariate analysis of PFS and OS. Cox proportional hazard model was used for multivariate analysis.

## Results

### Demographic details and baseline evaluation (“Table 1”)

A total of 94 patients satisfied the predefined selection criteria. The median age of the cohort was 51 years (Range 27–75 years). The cohort was male predominant, with 53 males (56.4%) and 41 females (43.6%). Eighteen patients (19.1%) were current or previous smokers while history of tobacco use was seen in 24 patients (25.5%). Comorbidities observed included hypertension in 11 (11.7%), diabetes mellitus type 2 in 8 (8.5%), liver dysfunction in 4 (4.3%) and cardiac dysfunction in 3 (3.2%) patients, respectively. The ECOG PS was 0–2 in 75 patients (79.8%), and 3–4 in 19 patients (20.2%). The median haemoglobin level was 11.4g/dl (IQR: 11.4–13.9 g/dl) and the median serum albumin level was 4g/dl (IQR: 3.5–5.2 g/dl).

**Table 1:** Demographic Data and baseline tumor characteristics.

Characteristic	Number (%)
Number of patients	94
Median age (years)	51 (range: 27–75)
Gender	
• Male	53 (56.4)
• Female	41(43.6)
Habits	
• Smokers (former and current)	18 (19.1)
• Tobacco users	24 (25.5)
Comorbidities	
• Hypertension	11 (11.7)
• Diabetes Mellitus	8 (8.5)
• Liver Dysfunction	4 (4.3)
• Cardiac Dysfunction	3 (3.2)
ECOG PS	
• 0	2 (2.1)
• 1	53 (56.4)
• 2	20 (21.3)
• >2	19 (20.2)
Stage	
• III	3 (3.2)
• IV	91 (96.8)
Number of metastatic sites	
• Median number of metastatic sites	2 (0–7)
• 1	24 (25.5)
• 2	29 (30.9)
• 3	23 (24.5)
• 4	9 (9.6)
• 5	3 (3.2)
• 7	1 (1.1)
Sites of metastases	
• Pleural effusion	42 (44.7)
• Opposite Lung	40 (42.6)
• Bone	48 (51.1)
• Liver	23 (24.5)
• Brain	21 (22.3)
• Cervical nodes	13 (13.8)
• Abdominal nodes	13 (13.8)
• Adrenal	6(6.4)
• Soft tissue	2 (2.1)
• Pericardial effusion	2 (2.1)
• Others	1 (1.1)
Location of disease	
• Intrathoracic only	24 (25.5)
• Extrathoracic only	2 (2.1)
• Intrathoracic and extrathoracic	68 (72.3)

### Tumor Characteristics (“Table 1”)

All patients 94 had adenocarcinoma or adenosquamous on histopathology of biopsy specimen. Ninety one patients (96.8%) patients were diagnosed with stage IV disease, while 3 patients had stage III disease. The median number of metastatic sites was 2 (range 0–7), with commonest sites of metastasis being pleural effusion in 42 (44.7%), opposite lung in 40 (42.6%), bone in 48 (51.1%), liver in 23 (24.5%) and brain in 21 (23.8%) patients, respectively. Disease was purely intrathoracic in 24 patients (25.5%), extrathoracic only in 2 patients (2.1%) and a combination of intra- and extra–thoracic disease was seen in the remainder of patients (72.3%).

### Distribution of first line treatment regimens (“S1 Table”)

Sixty patients (63.8%) received chemotherapy as first line treatment, 21 patients (22.3%) were started on Crizotinib upfront, while 10 patients (10.6%) received EGFR TKIs and 3 patients (3.2%) were planned for best supportive care only. The latter 2 categories were started on their respective treatment prior to receipt of ALK positive status.

Chemotherapeutic regimens used upfront were Pemetrexed–Platinum, Gemcitabine–Platinum and Paclitaxel-Platinum in 52 (86.6%), 5 (8.3%) and 3 (5%) patients, respectively. Overall, of the 94 patient cohort, 69 patients (73.3%) were exposed to Crizotinib at some point of time, while 25 patients (26.6%) never received Crizotinib.

### Logistic considerations associated with exposure to Crizotinib (“Table 2”)

The reasons for patients not being started on Crizotinib on diagnosis were disease burden and being symptomatic necessitating emergent treatment in 17 patients (23.3%), ALK testing not done initially in 17 patients (23.3%), lack of initial financial feasibility in 16 patients (21.9%), delay in final reporting of ALK status (12.3%), patient unwillingness to wait for reports (5.5%), previous treatment in the curative setting (1.4%) and ALK testing done on progression only (1.4%). Reasons were unavailable in 8 patients (11%).

**Table 2:** Logistic considerations associated with exposure to Crizotinib.

Characteristic	Number (%)
Reasons for not taking Crizotinib upfront (n = 73)	
• Symptomatic, requiring urgent treatment	17 (23.3)
• ALK testing not done upfront	17 (23.3)
• Lack of initial financial feasibility	16 (21.9)
• Delay in final reporting/availability	9 (12.3)
• Patient unwilling to wait for reports	4 (5.5)
• Previously treated in the adjuvant setting	1 (1.4)
• ALK testing on progression	1 (1.4)
• Reason not available	8 (11)
Reasons for lack of exposure to Crizotinib ever (n = 25)	
• Lack of financial feasibility	17 (68)
• Best supportive care upfront	7 (28)
• Not known	1 (4)
Reasons for shifting to Crizotinib (n = 48)	
• Shifted on receipt of ALK testing positivity and financial support	26 (54.1)
• Progression on chemotherapy	16 (33.3)
• Maintenance	6 (12.5)
Procurement of Crizotinib (n = 69)	
• Self, no credit	22 (31.8)
• Self, credit	9 (13)
• Sponsored by NGO (Non- Governmental Organization)	38 (55)

Besides patients receiving Crizotinib as first line therapy, a further 48 patients (51%) received Crizotinib at a later point in their treatment course. Reasons for shifting to Crizotinib from prior therapy were receipt of ALK positive status in 26 patients (54.1%), progression on previous treatment in 16 patients (33.3%) and Crizotinib considered as maintenance post response /stabilization of disease after 3–6 cycles of chemotherapy in 6 patients (12.5%).

Assessment of method of procurement of Crizotinib showed that 31 patients (44.8%) gained access via methods of self-payment. Of these patients, 22 patients (31.8%) purchased Crizotinib by self-financing (including insurance), while the remaining 9 patients (13%) were government supported as part of their employment package. The majority of patients, 38 (55%), received Crizotinib free of cost via support from the Non-governmental Organizations’ (NGOs’).

### Safety and adverse events with Crizotinib (“Table 3”)

Grade 3/4 adverse events along with other significant toxicities are reported. The common side effects seen in patients include visual hallucinations (26.1%), grade 3/4 anemia (20.3%), and elevation of AST/ALT (8.6%), grade 3/4 vomiting (7.2%), grade 3/4 diarrhoea (4.3%), ECG abnormalities (4.3%) and grade 3/4 fatigue (4.3%). Other rare side–effects included renal insufficiency and Crizotinib induced pneumonitis in 1 patient (1.4%) each.

**Table 3:** Adverse Events and safety.

Characteristic	Number (%)
Adverse events	
• Visual hallucinations	18 (26.1)
• Anemia (Grade 3/4)	14 (20.3)
• AST/ALT elevation (Grade 3/4)	6 (8.6)
• Vomiting (Grade 3/4)	5 (7.2)
• Neutropenia	3 (4.3)
• Diarrhoea (Grade 3/4)	3 (4.3)
• ECG abnormalities	4 (5.7)
• QTc prolongation	3
• Symptomatic sinus bradycardia	1
• Fatigue (Grade 3/4)	3 (4.3)
• Interstitial pneumonitis	1 (1.4)
• Renal insufficiency (Grade 3/4)	1 (1.4)
• Mucositis	0
• Thrombocytopenia	0
Drug interruptions	20 (29)
• Toxicity	17 (85)
• Drug unrelated	2(10)
• Lack of compliance	1 (5)
• Median duration of drug interruption (days)	7 (range 2–90)
Drug interruptions caused by toxicity (n = 17)	
• AST/ALT elevation (Grade 3/4)	6 (35.2)
• ECG abnormalities	2 (11.7)
• Vomiting (Grade 3/4)	2 (11.7)
• Interstitial pneumonitis	1 (5.8)
• Acute renal insufficiency (Grade 3/4)	1 (5.8)
• Visual hallucinations	1 (5.8)
• Fatigue (Grade 3/4)	1 (5.8)
• Neutropenia (Grade 3/4)	1 (5.8)
• Combination of Grade 2 adverse events	2 (11.7)
Dose reduction post drug resumption (n = 20)	
• Dose reduction	8 (40)
• Full dose	12 (60)

Twenty patients (29%) out of 69 required temporary cessation of Crizotinib for a median of 7 days (range: 2–90 days). Drug toxicity, commonly AST/ALT elevation (6 patients), ECG abnormalities (2 patients), and vomiting (2 patients), caused temporary Crizotinib cessation in 17 patients (85%), while drug unrelated causes in 2 patients and lack of compliance in 1 patient required stoppage of Crizotinib.

Crizotinib was resumed at full doses in 12 patients (60%) on restarting, while 8 patients (40%) required starting at lower doses. 4 of these patients underwent dose escalation to full doses later.

### Response rates and outcomes (“Tables 4–6”)

Response rates are reported for patients on Crizotinib. Partial response was seen in 37 patients (53.6%), Stable Disease in 13 (18.8%), with progressive disease in 2 patients (2.8%). Eight patients could not be evaluated by RECIST, while 9 (13%) patients are yet to undergo response evaluation.

**Table 4:** Response rates, Survival and pattern of progression on Crizotinib.

Characteristic	Number (%)	p value
Type of response to Crizotinib (n = 69)		
• Partial response (PR)	37(53.6)	
• Stable Disease (SD)	13 (18.8)	
• Progressive (PD)	2 (2.8)	NA
• Evaluation awaited	9 (13)	
• Could not be evaluated	8 (11.5)	
• Disease Control rate (DCR = CR+PR+SD)	50 (72.4)	
Outcomes		
➢ Median PFS (months)	10	
• Crizotinib upfront	12 (7.5–16.4)	0.159
• Crizotinib later	10 (6.8–13.1)	
➢ Median OS (months)	Not reached	
○ Crizotinib upfront	Not reached	0.502
○ Crizotinib later	39.8 (5.24–74.48)	
➢ 1 year OS (%)	81.2	
Characteristics of disease progression (n = 15)		
• Local only	3 (20)	
• Local & Distant	8 (53.3)	NA
• Distant only	3 (20)	
• Not available	1 (6.7)	
Progression with relation to brain metastases (n = 15)		
• Not progressed in brain	8(53.3)	
• Progression in brain only	4 (26.7)	NA
• Progression in brain and others sites	2 (13.3)	
• Not available	1 (6.7)	

**Table 5:** PFS and Prognostic factors.

Variable	Median PFS in months(95% CI)	HR (95%CI)	p value
**Age (n = 94)**		1.029 (0.458–2.311)	NS
Age <60 years:	12 (9.7–14.2)		
Age > or = 60 years	9 (3.6–14.3)		
**ECOG PS (n = 94)**		4.07 (1.82–9.13)	0.001
0–2	10 (8.7–13.2)		
>2	1.5 (0.9–2.0)		
**Exposure to Crizotinib (n = 94)**		2.16 (1.09–4.26)	0.026
Yes	10 (7.7–12.2)		
No	2 (0.8–3.1)		
**Presence of brain metastases (n = 94)**		1.205 (0.56–2.58)	NS
No	10 (5.8–14.2)		
yes	7 (1.4–12.5)		

NS–Not significant.

**Table 6:** OS and Prognostic factors.

Variable	Median OS in months(95% CI)	HR (95%CI)	p value
**Age (n = 94)**		0.93 (0.27–3.69)	NS
Age <60 years:	Not reached		
Age > or = 60 years	39.86 (2.2–77.4)		
**ECOG PS (n = 94)**		34.18 (8.89–131.3)	<0.001
0–2	Not reached		
>2	2.96 (1.30–4.62)		
**Exposure to Crizotinib (n = 94)**		6.54 (2.02–21.13)	0.002
Yes	39.86 (5.29–74.43)		
No	11.2 (0.0–28.50)		
**Presence of brain metastases (n = 94)**		2.30 (0.69–7.69)	NS
No	Not reached		
Yes	39.86 (5.29–74.43)		

NS–Not significant.

During the period of analysis, 15 patients progressed on Crizotinib. Their pattern of progression is shown in Table 5. 8 patients (53.3%) had local and distant progression, while 3 patients (20%) each had local only distant only progression. 6 patients (40%) progressed in the brain, of which 4 (26.7%) had new onset metastases or progression in the brain only. 8 patients (53.3) with progression at other sites did not progress in the brain.

**Fig 2:**
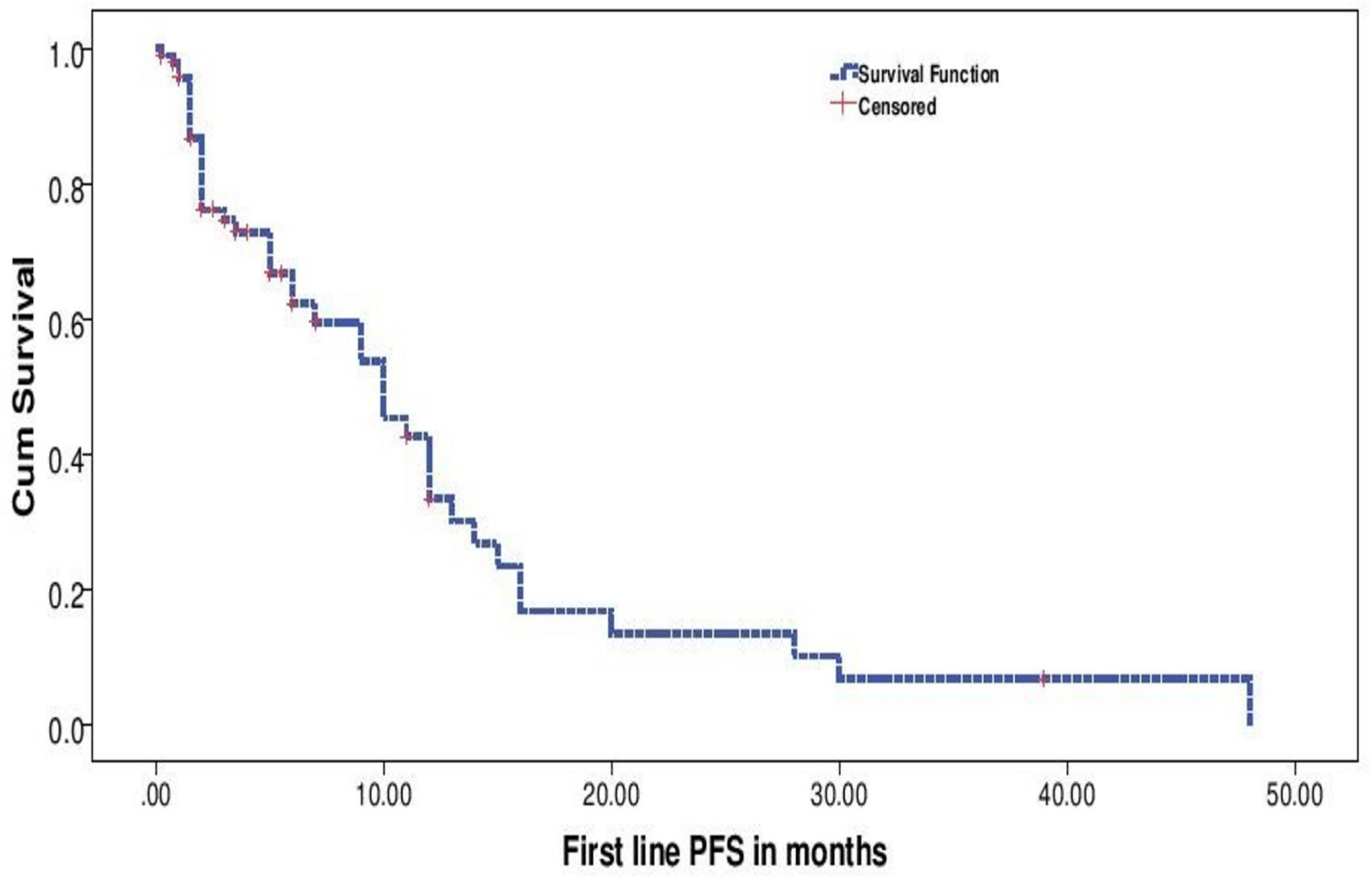
Overall Progression Free Survival.

**Fig 3:**
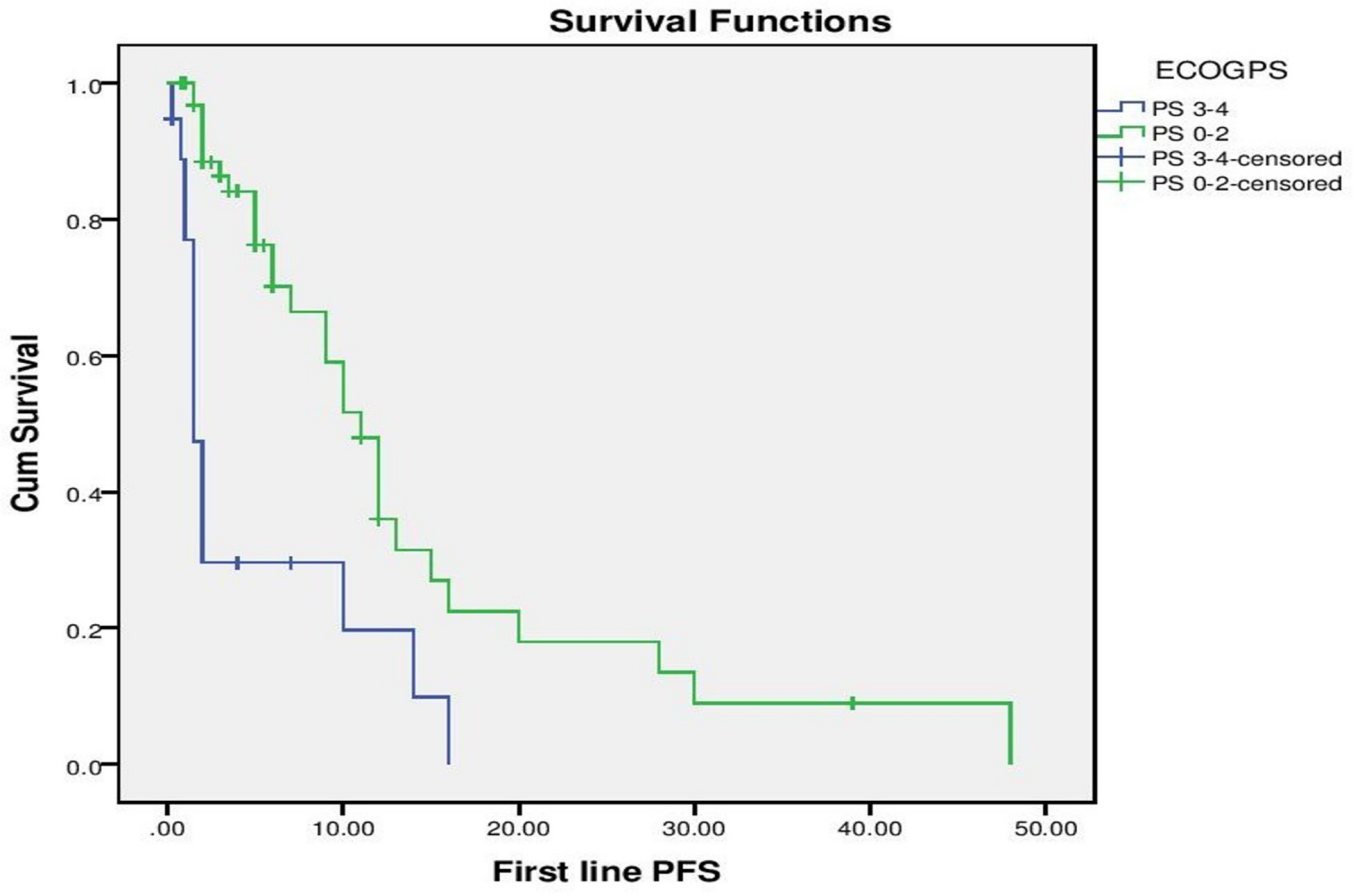
Progression Free Survival as per Performance Status.

**Fig 4:**
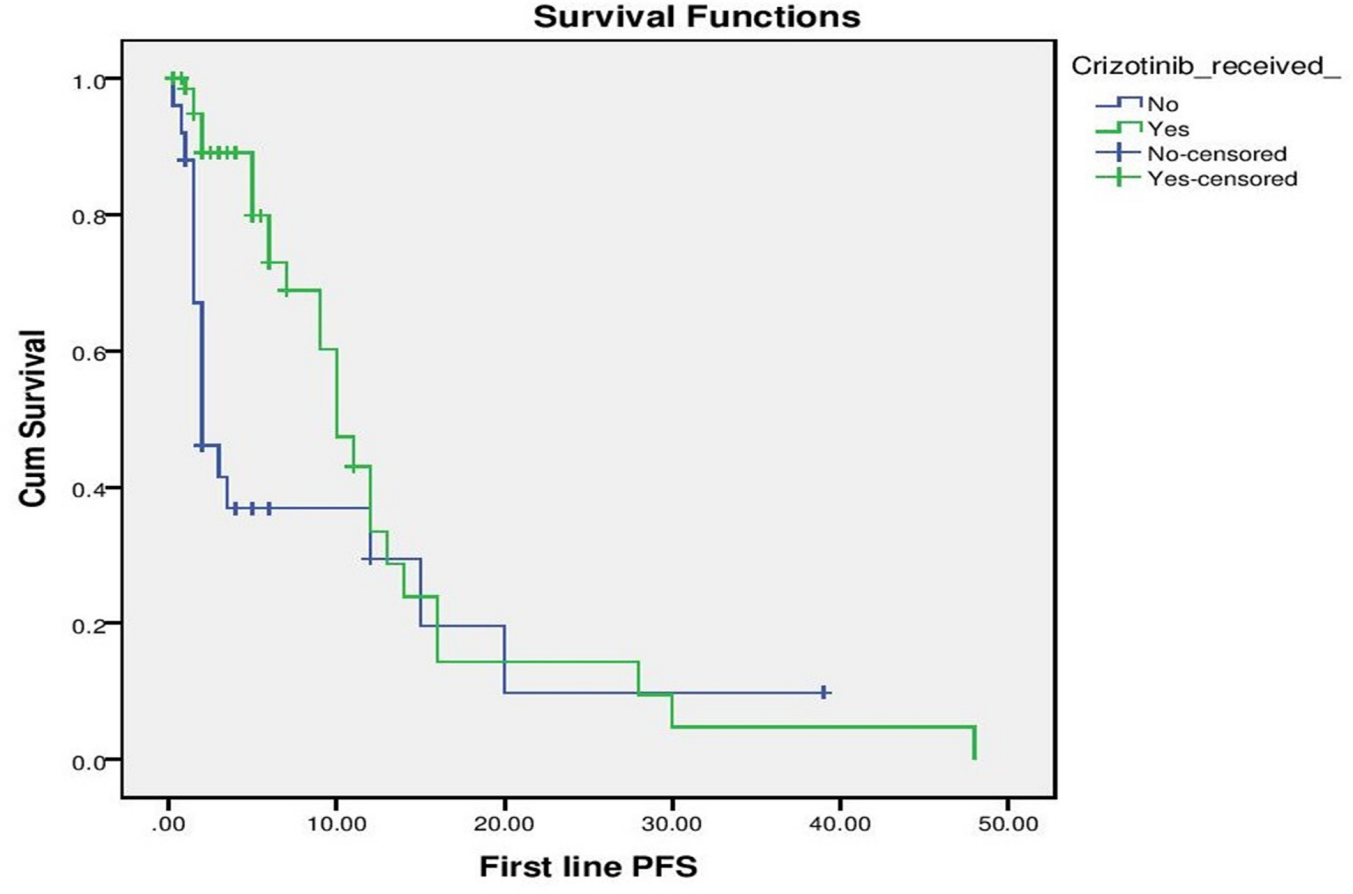
Progression Free Survival as per exposure to Crizotinib.

With a median follow up of 9 months for the entire cohort, median PFS for the entire cohort was 10 months (“Fig 2”). On evaluation of prognostic factors, on univariate analysis, ECOG PS 0–2 predicted for a significantly better PFS than ECOG PS >2 (10 months vs. 1.5 months, p <0.001) and exposure to Crizotinib versus no exposure to Crizotinib predicted for significantly longer PFS (10 months vs. 2 months, p = 0.028). Multivariate analysis confirmed that ECOG PS (p = 0.001, HR– 4.07, 1.821–9.138, 95% CI) and exposure to Crizotinib (p = 0,026, HR 2.16, 1.098 vs. 4.269, 95% CI) predicted for longer PFS (“Figs 3 and 4”).

**Fig 5:**
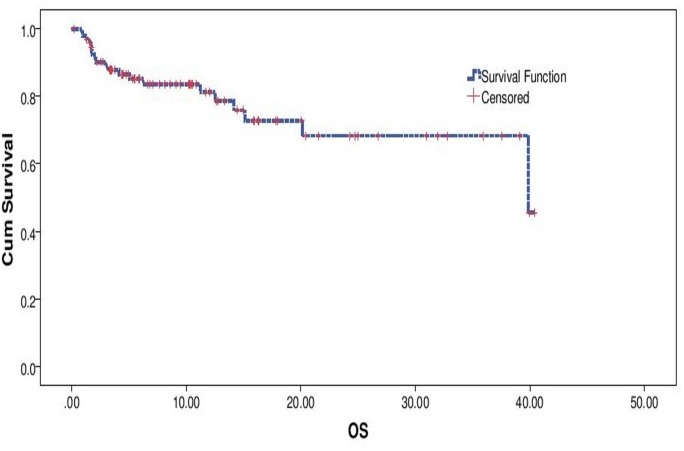
Overall Survival.

**Fig 6:**
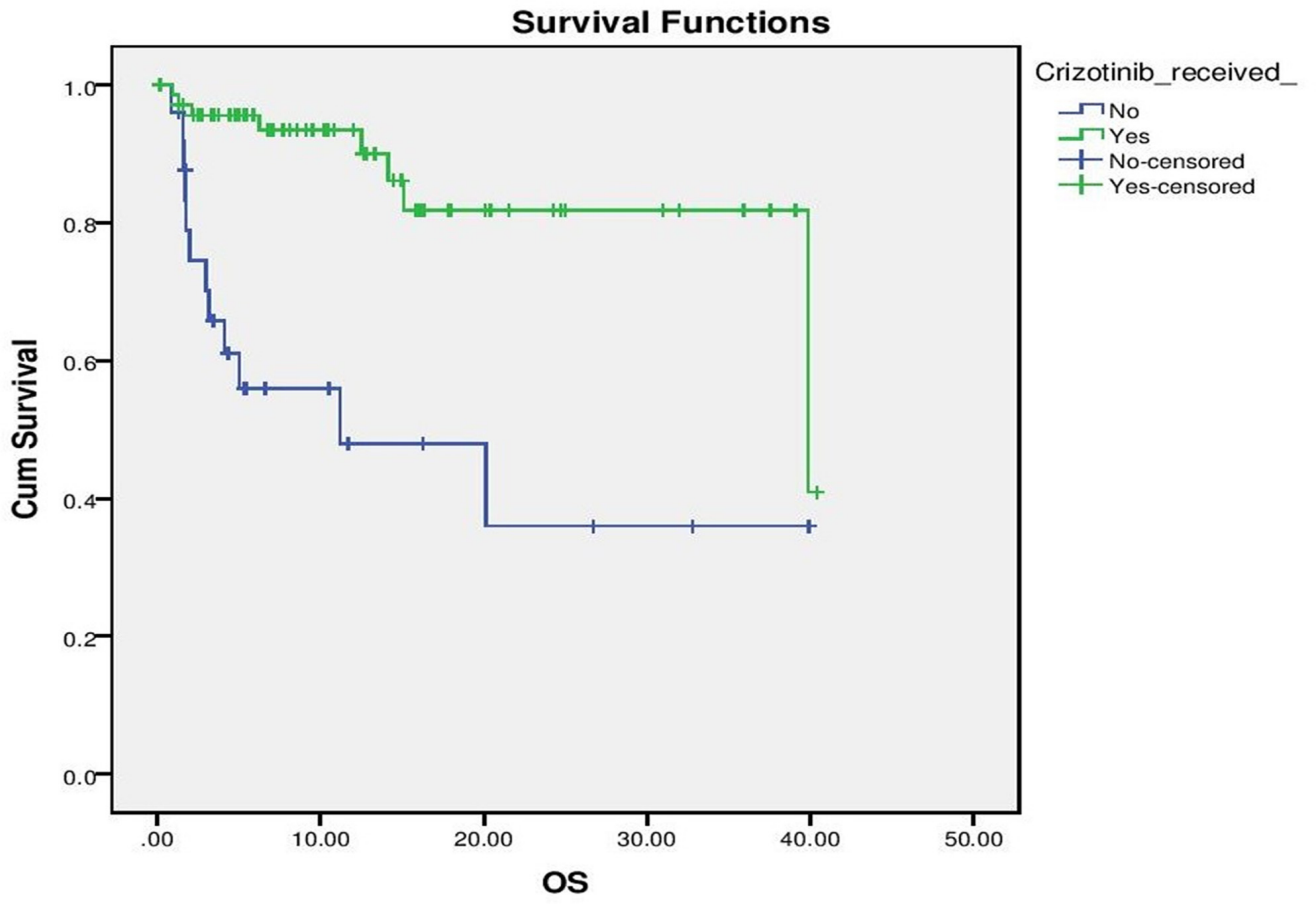
Overall Survival as per Performance Status.

**Fig 7:**
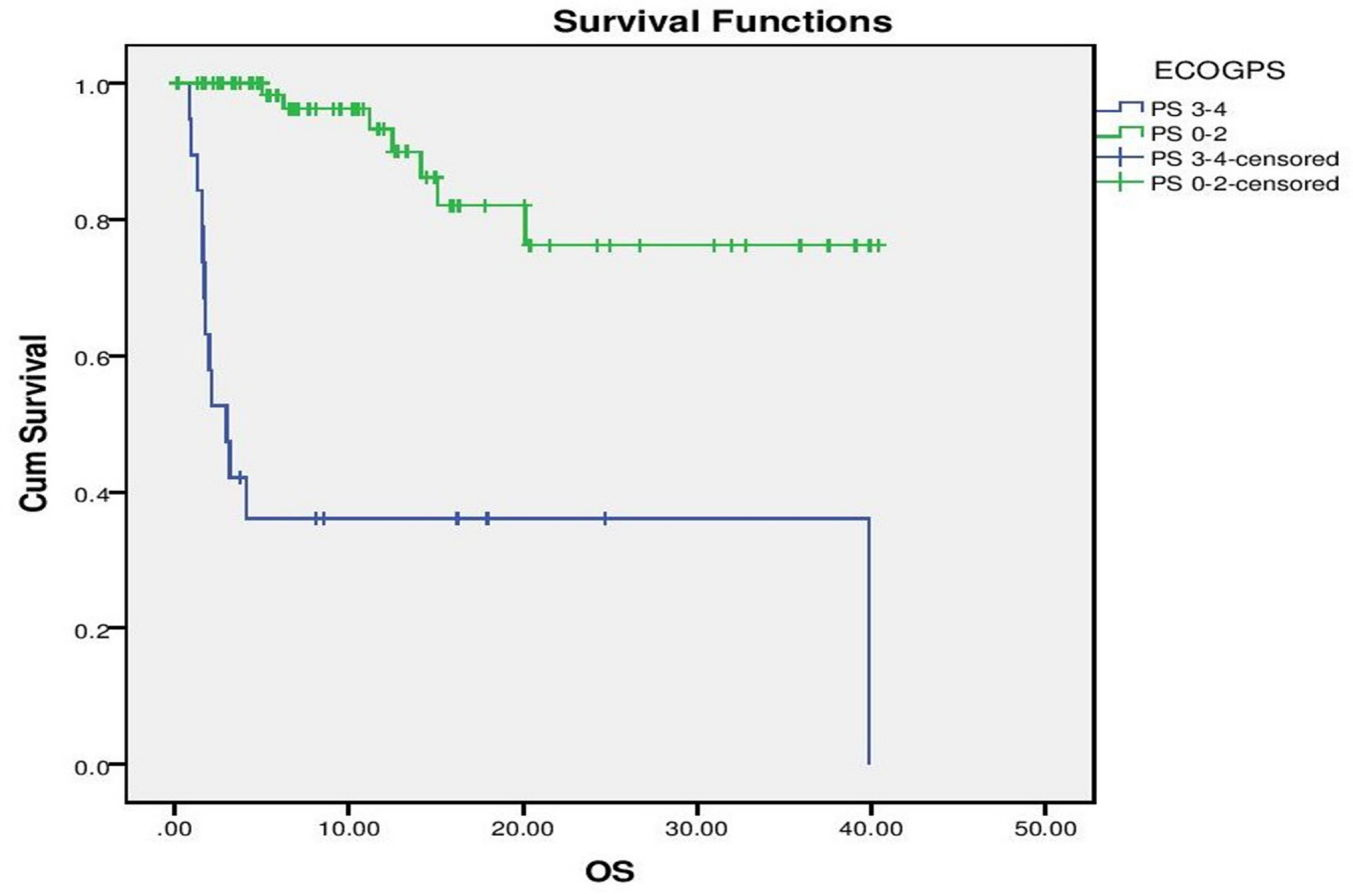
Overall Survival as per exposure to Crizotinib.

Median OS was not reached for the entire cohort, with estimated 1 year survival being 81.2% (“Fig 5”). An ECOG PS 0–2 versus ECOG PS >2 (Median OS not reached vs. 2.967 months, p<0.001) and exposure to Crizotinib versus non exposure to Crizotinib (Median OS 39.86 vs 11.2, p<0.001) predicting for significantly longer OS. These factors retained their prognostic ability on multivariate analysis (ECOG PS 0–2 vs. PS >2, p<0.001, HR- 34.18, 8.898–131.307, 95% CI and Crizotinib vs. Non Crizotinib, p = 0.002, HR 6.54, 2.026–21.136, 95% CI) (“Figs 6 and 7”)

## Discussion

The current era of targeted therapy and precision medicine has ushered in a plethora of targeted agents which have shown impressive results in trial settings. However, reproducibility in the real world population is an increasing concern. The applicability of trial data to a non trial population has to take into account the greater incidence of co-morbidities, less stringent monitoring, a more heterogeneous populace and most importantly, issues of access to potentially expensive newer molecules. It is in this scenario that this study assumes importance where besides examining outcomes with Crizotinib, we have attempted to gauge practise patterns associated with the use of this drug as well as throw light on its accessibility to a financially constrained population.

The demographic profile and tumor characteristics of our patients was similar to that seen in trials and other large retrospective analysis [4,10,11], with the exception of a slight male predominance and lower incidence of smokers seen in our study. We did not differentiate between former smokers and current smokers in this analysis, though there is evidence of higher incidence of ALK fusion positivity in non-smokers and former smokers versus current smokers [12,13].

A major focus of this study was outlining the treatment patterns in ALK positive NSCLC. A majority of patients were started on chemotherapy (63.8%) upfront, and then later shifted to Crizotinib, such that a significant percentage (73.3%) were exposed to Crizotinib on the whole. Lack of initial exposure to Crizotinib did not seem to affect PFS significantly, as long as patients did get exposed to Crizotinib in their treatment course. One of the potential reasons for this lack of difference is the suggested preferential activity of Pemetrexed in ALK positive NSCLC [14,15], which was also the agent most commonly used as first line therapy in our cohort. However, exposure to Crizotinib is clearly essential in terms of outcomes and this was borne out by the significantly increased PFS and OS compared to patients never exposed to Crizotinib (PFS—10 months vs. 2 months and estimated OS– 39.86 months vs. 11.2 months).

The major reasons ascertained for not starting Crizotinib upfront were symptomatic disease requiring urgent treatment, ALK testing not done upfront, lack of initial financial feasibility and a delay in finalization/reporting of ALK positive status. The latter reasons are a reflection of the logistics and financial constraints involved in treating this cohort in the real world with Crizotinib. ALK testing by break-apart FISH probes is an expensive test for the majority of our population and although IHC (less expensive compared to testing by FISH) has shown good concordance with FISH [16–18], it has been only recently been approved for ALK testing and its use in our institution has been standardized from mid-2014 only.

There has been considerable debate regarding the cost effectiveness of ALK testing itself and first line therapy with Crizotinib. A Canadian study examined this very issue and concluded that testing for EML4-ALK in stage IV NSCLC and the subsequent treatment with Crizotinib was not cost effective due to the cost of Crizotinib as well as the low frequency of EML4-ALK in the general population [19]. This is a pertinent point as even if the costs of diagnostic methods are reduced, there remains the larger question of testing for a niche targetable mutation and more importantly, affordability of therapy. The monthly subsidized cost of standard dose Crizotinib in our institution is Rs 76000 (US$ 1120), which is beyond the reach of the majority of our population. It is also to be recognized that the patient population treated in our institution consists of patients in both the private and public setting. Despite consisting a cohort of patients from a potentially affordable private setting, a majority of patients in this study were unable to start Crizotinib on receipt of reports. This is reflected in our practise as we were able to start Crizotinib upfront in only 22.3% of patients. However, what is heartening is that during the treatment course of our ALK positive patient cohort, we were able to expose a further 51% to Crizotinib. This was majorly feasible due to the active financial support provided by NGOs’ in procuring the drug. Indeed, 55% of our patient population gained access to Crizotinib via this mechanism, with a further 13% receiving Crizotinib due to employer support.

Within the confines of the above constraints, our study confirms the favorable outcomes and tolerability of Crizotinib in the Indian population. A PFS of 10 months, which is commiserate with that seen in the pivotal PROFILE 1014 study [4], and higher than that seen in the trial using Crizotinib as second line therapy by Shaw et al [10], was observed. The response rates of 53.6% in this study appear lower than the previous quoted studies, partly due to 9 (13%) patients awaiting response evaluation and a further 8 patients (11.5%) who could not have responses assessed by RECIST, despite clinically significant response. Median OS was not reached for the entire population, with an estimated 1 year survival rate of 81.2%. ECOG PS > 2 was a significant negative predictor for EFS and OS, reiterating the importance of PS in prognosticating lung cancer patients [20,21]. However, while the registration trials for Crizotinib have systematically excluded patients with poor ECOG PS, there is some early evidence in the form of case reports and studies which suggests that even patients with a poor ECOG PS may have dramatic benefits after initiation of targeted therapy. Such an approach, directed at treating patients with poor PS, has also been seen in EGFR activating mutation positive NSCLC treated with Erlotinib and highlights the unique ability of these effective drugs to be explored in this setting in the clinic[22–24]

The profile of adverse events in our patients, while stressing on Grade 3/4 toxicities, seemed higher than quoted in trial data, especially with respect to anemia (20.3%) and ECG abnormalities (5.7%), with lesser frequencies of other adverse events. Although 29% of our patients required dose interruptions, the median duration of cessation was for 1 week, with a majority able to resume Crizotinib at full doses.

Our study, while delving into the patterns of treatment of ALK positive NSCLC has certain limitations. The retrospective nature of the study, potential underreporting of subjective adverse events like fatigue, etc. and short duration of median follow-up are lacunae that affect the impact of this study.

In conclusion, the treatment of ALK positive NSCLC in the Indian population has its own set of challenges, predominantly socio- economic in nature. Exposure to Crizotinib and ECOG PS appear to be significant predictors of outcomes. However, with active extramural support, a majority of patients are getting exposed to Crizotinib with clinically relevant efficacy, outcomes and tolerability similar to published international data.

## Supporting Information

S1 Table “Initial treatment distribution”. (DOCX)
