# Correction: ALK Positive Lung Cancer: Clinical Profile, Practice and Outcomes in a Developing Country

**DOI:** 10.1371/journal.pone.0168221

**Published:** 2016-12-09

**Authors:** The PLOS ONE Staff

## Notice of Republication

This article was republished on September 22, 2016, as two Supporting Information files were published in error. The publisher apologizes for the errors. Please download this article again to view the correct version.
