# Supplementary material for: ALK Positive Lung Cancer: Clinical Profile, Practice and Outcomes in a Developing Country
Source: PLoS One. 2016 Sep 16;11(9):e0160752. doi: 10.1371/journal.pone.0160752 (PMC5026380; doi:10.1371/journal.pone.0160752)
Supplement: S1 Table — (DOCX) [file pone.0160752.s001.docx]

Table S1. Initial treatment distribution

| Characteristic | Number (%) |
| --- | --- |
| First line treatment   - Chemotherapy - Crizotinib - EGFR tyrosine kinase inhibitor - Best supportive care | 60 (63.8)  21 (22.3)  10 (10.6)  3 (3.2) |
| Chemotherapy regimens (n=60)   - Pemetrexed – platinum - Paclitaxel – platinum - Gemcitabine - platinum | 52 (86.6)  5 (8.3)  3 (5) |
| Exposure to Crizotinib   - Crizotinib upfront - Crizotinib during later course of treatment - Not exposed to Crizotinib during treatment | 21 (22.3)  48 (51)  25 (26.6) |
